# Vaginal NOTES adnexal surgery: Results of a retrospective cohort study in The Netherlands

**DOI:** 10.1016/j.eurox.2025.100422

**Published:** 2025-08-05

**Authors:** Rebecca Henschen, Anouk J.M. Bus, Nicol A.C. Smeets, Marlies Y. Bongers, Martine M.L.H. Wassen

**Affiliations:** a Department of Obstetrics and Gynaecology, Zuyderland Medical Centre, Henri Dunantstraat 5, Heerlen 6419 PC, the Netherlands; b GROW School for Oncology and Reproduction, Maastricht University Medical Centre, Maastricht, the Netherlands; c Maastricht University Medical Centre, Maastricht, the Netherlands

**Keywords:** Adnexal surgery, Benign, Laparoscopy, VNOTES

## Abstract

**Objective:**

Vaginal natural orifice transluminal endoscopy (vNOTES) is a minimally invasive technique gaining popularity for several gynaecological procedures. This study presents the first 81 adnexal cases, performed in The Netherlands.

**Design, setting, participants, and intervention:**

This retrospective cohort study included patients who underwent vNOTES adnexal surgery for benign indications at Zuyderland Medical Centre between March 2020 and August 2024. Baseline characteristics, surgical outcomes, and per- and postoperative outcomes were analysed. Two expert vaginal and endoscopic gynaecological surgeons performed all procedures.

**Results:**

A total of 81 patients underwent adnexal surgery using vNOTES. Indications were definitive contraception (60.5 %; n = 49), ovarian cysts (18.5 %; n = 15), risk-reducing surgery for gene mutation carriers (16.0 %; n = 13), ectopic pregnancy (3.7 %; n = 3), and request for artificial menopause due to complaints (1.2 %; n = 1). Procedures performed were bilateral salpingectomy (60.5 %; n = 49), bilateral salpingo-oophorectomy (28.4 %; n = 23), unilateral salpingo-oophorectomy (4.9 %; n = 4), unilateral salpingectomy (3.7 %; n = 3), unilateral ovariectomy (1.2 %; n = 1), and unilateral salpingo-oophorectomy with unilateral salpingectomy (1.2 %; n = 1). The mean surgical time was 38.7 min (SD 17.9 min), with a mean blood loss of 26 mL (SD 42.4 mL). There was one (1.2 %) conversion to laparoscopy, and two (2.5 %) intra-operative complications without re-interventions. Most patients (88.8 %) were treated in a day-care setting. Four postoperative complications (4.9 %) were reported within six weeks after surgery.

**Conclusion:**

This study shows that vNOTES is a safe and feasible, less invasive and scarless alternative to laparoscopic and open surgery for benign adnexal pathology. More evidence is needed to compare vNOTES adnexal surgery with laparoscopy.

**Summation:**

vNOTES is a safe and feasible, less invasive alternative without abdominal scars compared to laparoscopic and open abdominal surgery for benign adnexal pathology.

## 1 Introduction

### 1.1 Background

In recent years, vaginal natural orifice transluminal endoscopic surgery (vNOTES) is an emerging minimally invasive surgical technique. More surgeons have been trained worldwide, and scientific evidence is increasing about the advantages of this technique. NOTES is a minimally invasive surgical technique that involves accessing the abdominal cavity through natural orifices, such as the vagina (vNOTES) [1]. The most performed procedure with vNOTES is the hysterectomy [2]. This is a safe and non-inferior alternative to conventional laparoscopy, without conversion to laparotomy [1]. Adnexal surgery using a transvaginal NOTES approach was first described in 2012 and has been increasingly used for several benign indications [3]. The NOTABLE trial showed that vNOTES is a safe and desirable approach for adnexectomy and is non-inferior compared to conventional laparoscopy with shorter operating times, less postoperative pain, reduced analgesic use, no conversions, and no scars [4]. Feasibility and safety of peritoneal access via transvaginal routes have been demonstrated for several adnexal indications [4], [5], [6], [7], [8]. In October 2023, the National Institute for Health and Care Excellence (NICE) stated that the available evidence is insufficient regarding the safety and efficacy of vNOTES adnexal surgery for benign gynaecological indications [9]. While the evidence includes one high-quality randomized controlled trial (RCT) for adnexectomy, the remaining evidence is of lower quality, attributed to study designs and small sample sizes [9]. Also, the IDEAL recommendations for surgical innovation states that a RCT is operationally undesirable and scientifically of limited use, due to procedural modifications and varying eligibility [10].

This retrospective cohort study was conducted to provide insights into the safety and feasibility of adnexal surgery using vNOTES. The primary aim was to determine the peri-and postoperative outcome after vNOTES adnexal surgery.

## 2 Methods

### 2.1 Study design, setting and participants

A retrospective cohort study was performed in Zuyderland MC, The Netherlands. Patients with a benign indication requiring adnexal surgery were counseled regarding different surgical approaches like conventional endoscopic surgery or vNOTES surgery and could choose their preferred method. Oral informed consent was obtained from all patients before inclusion. Women scheduled for vNOTES adnexal surgery between March 2020 and August 2024 were included in the study. The sample size was not determined prior to data collection because it concerns a descriptive cohort study.

Eligible patients scheduled for vNOTES adnexal surgery had a benign indication for the procedure and no exclusion criteria for vNOTES. Exclusion criteria were a history (or suspicion) of endometriosis, rectal surgery, pelvic inflammatory disease or pelvic radiation.

Baseline characteristics, perioperative- and postoperative outcomes were recorded and analysed. All procedures were performed by two experienced endoscopic gynaecological surgeons (NS and MW), with expertise in conventional laparoscopy and vaginal surgery. MW and NS performed respectively 16 and 31 vNOTES hysterectomies before starting vNOTES adnexal surgery.

#### 2.1.1 Treatment protocol

All women received 2 g of cefazolin and 500 mg of metronidazole 30 min before surgery. Patients were positioned in lithotomy position and received general anaesthesia. After disinfection and sterile draping, the bladder was emptied using a Foley catheter or a single-use catheter, depending on the indication and estimated surgery duration. vNOTES adnexal surgery consisted of three phases:

#### 2.1.2 Surgical procedure

##### 2.1.2.1 Phase 1

**Fig. 1:**
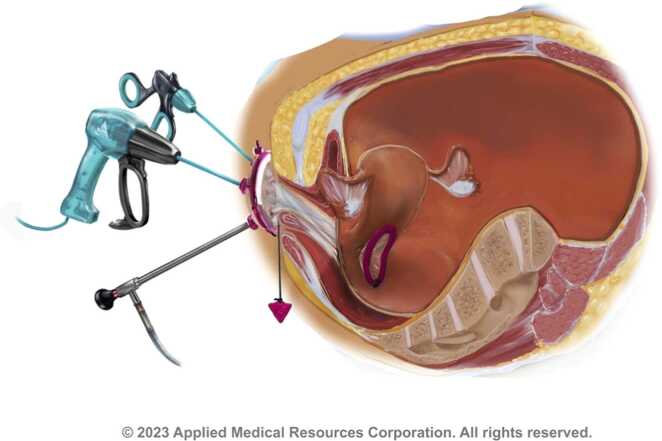
Representation of vNOTES adnexal surgery [11].

A posterior colpotomy of 2.5 cm was performed after infiltrating with 40 mL of 0.5 % adrenaline ropivacaine. The rectouterine pouch was opened using cold scissors, followed by insertion of the Alexis Wound retractor (Applied Medical). The inner ring was pushed into the peritoneal cavity, initially using a Breisky retractor and later the introducer (Applied Medical). The Breisky retractor or introducer was removed, and the outer ring of the Alexis was rolled inwards twice. Three trocars were inserted in the 7 cm GelPOINT V-Path Mini, and the V-Path was connected to the outer ring of the Alexis. The surgical procedure was initiated by introducing a 30° vision port camera, a grasper, and a sealing device (Fig. 1). A pneumoperitoneum was created with a preferred maximum pressure of 10 mmHg.

##### 2.1.2.2 Phase 2

The patient was positioned in a 20-degree Trendelenburg position. The abdominal cavity was inspected, and the planned adnexal surgical procedure was performed. After achieving haemostasis, instruments were removed.

##### 2.1.2.3 Phase 3

The GelPOINT V-Path Mini was removed, specimen extraction and Alexis Wound retractor was removed by pulling on the string and squeezing the inner ring. The patient was positioned in a 10-degree Trendelenburg position. The posterior colpotomy was closed with three Vicryl 2–0 sutures, and haemostasis was achieved. The Foley catheter was removed at the end of the procedure if applicable. Patients received post-operative analgesia according to the local pain protocol, with opioids avoided when possible. Depending on the post-operative clinical condition, the surgery end time, patients’ preference, and medical history, patients were discharged on the same day.

### 2.2 Variables and data measurements

The pre-operative patient characteristics that were collected included: age, body mass index (BMI), medical history, parity and surgical indication.

Intra-operative the following data were collected: total surgical time, duration of the three individual phases of vNOTES, technique conversion, blood loss (mL), and intra-operative complications.

Post-operative the following data were collected: procedures performed with same-day discharge (SDD), and complications in the first six postoperative weeks, classified by Clavien-Dindo [12].

### 2.3 Statistical analysis

Statistical analysis was performed using IBM SPSS Statistics version 25 (SPSS, NY, USA). Pre-, per- and post-operative data were collected and analysed with univariate descriptive analysis. Ordinal variables were presented as mean (range), nominal variables as frequencies (percentage) and continuous variables as mean (standard deviation (SD)). In Table 3, the Shapiro-Wilk Test assessed normal distribution within the two groups. Normally distributed variables are reported as mean (± SD), with p-values calculated by the Independent Samples t-Test. Non-normally distributed variables are presented as median (IQR), with p-values calculated by the Mann–Whitney *U* test. A significance level α (family-wise error rate) of 0.05 was used.

## 3 Results

From March 2020 to August 2024, 81 patients were included in this retrospective cohort study. Table 1 summarizes patient characteristics.

**Table 1:** Baseline characteristics.

	**Cohort** **(n = 81)**
Age (years)	42.3 (±12.7; 20–86)
BMI (kg/m^2^)	26.8 (±5.6; 19.1–43.3)
Previous abdominal surgery	25 (30.9 %)
*Laparoscopic surgery*	11 (44 %)
* Cholecystectomy*	3 (12 %)
* Appendectomy*	3 (12 %)
* Bariatric bypass surgery*	2 (8 %)
* Tubal ligation*	1 (4 %)
* Procedure unknown*	1 (4 %)
* ≧ 2 of the above*	2 (8 %)
*Open appendectomy*	1 (4 %)
*Caesarean section*	13 (52 %)
**Parity, n (%)**	
Nulliparous	5 (6.2 %)
Vaginal delivery	63 (77.8 %)
Caesarean section	7 (8.6 %)
Vaginal and caesarean section	6 (7.4 %)
**Indication for adnexal surgery, n (%)**	
Definitive contraception	49 (60.5 %)
Ovarian cyst	15 (18.5 %)
* < 5 cm*	3 (20.0 %)
* > 5 cm*	12 (80.0 %)
Risk reducing surgery because of gene mutation	13 (16.0 %)
Ectopic pregnancy	3 (3.7 %)
Request for artificial menopause because of complaints	1 (1.2 %)
**Surgical procedure, n (%)**	
Bilateral salpingectomy	49 (60.5 %)
Bilateral salpingo-oophorectomy	23 (28.4 %)
Unilateral salpingo-oophorectomy	4 (4.9 %)
Unilateral salpingectomy	3 (3.7 %)
Unilateral ovariectomy	1 (1.2 %)
Unilateral salpingo-oophorectomy with unilateral salpingectomy	1 (1.2 %)

BMI = Body Mass Index

### 3.1 Baseline characteristics

The mean age was 42.3 years (range 20–86 years), with a mean BMI of 26.8 kg/m^2^ (SD 5.6). In this cohort 6.2 % (n = 5) of the women were nulliparous, 77.8 % (n = 63) had at least one previous vaginal delivery, 8.6 % (n = 7) had one caesarean section and 7.4 % (n = 6) had a history of at least one vaginal delivery and one caesarean section. Additionally, 30.9 % (n = 25) had previous abdominal surgery (Table 1).

Adnexal surgery procedures were performed for the following indications: definitive contraception (60.5 %; n = 49), ovarian cysts (18.5 %; n = 15), risk-reducing surgery for gene mutation carriers (16.0 %; n = 13), (hemodynamically stable) ectopic pregnancy (3.7 %; n = 3), and request for artificial menopause due to complaints (1.2 %; n = 1). Procedures performed were bilateral salpingectomy (60.5 %; n = 49), bilateral salpingo-oophorectomy (28.4 %; n = 23), unilateral salpingo-oophorectomy (4.9 %; n = 4), unilateral salpingectomy (3.7 %; n = 3), unilateral ovariectomy (1.2 %; n = 1), and unilateral salpingo-oophorectomy with unilateral salpingectomy (1.2 %; n = 1) (Table 1).

Mean ovarian cyst size was 8.5 centimeters (range 3.6–23 centimeters). The dimensions of the cysts were determined through the pathology report postoperatively. No spillage occurred during surgery in this study. All adnexal cysts were histologically benign.

In five cases (6.2 %) an additional procedure was performed; three endometrial ablations, one transobturator tape placement and one Le Fort colpocleisis.

### 3.2 Surgical outcome

The mean total surgical time, excluding the case of conversion, was 38.7 min (SD 17.9). Mean durations of the individual phases of vNOTES surgery were: 8.2 min (SD 5.1) for phase 1, 21.2 min (SD 15.2) for phase 2, and 9.7 min (SD 6.9) for phase 3. The mean blood loss was 26 mL (SD 42.4), with a mean amount of CO_2_ used during surgery of 33 L (SD 30.0), and a mean intra-abdominal pressure of 9.9 mmHg (range 8–12 mmHg).

As described in Table 2, there was one conversion to laparoscopy (patient 32, the 19th patient operated on by the surgeon). This patient was 53 years old, with a BMI of 26 kg/m², vaginal parity, and no previous abdominal surgery. The indication was a risk-reducing bilateral salpingo-oophorectomy due to a BRCA2 gene mutation. The conversion was necessary because access to the rectouterine pouch was not possible. No adhesions in the rectouterine pouch were observed during laparoscopy.

**Table 2:** Surgical outcomes.

	**Cohort** **(n = 81)**
**Intra-operative outcomes**	
Mean total duration of surgery, minutes (SD)	38.7 (±17.9)*
* Phase 1*	8.2 (±5.1)*
* Phase 2*	21.2 (±15.2)*
* Phase 3*	9.7 (±6.9)*
Blood loss, mean (mL)	26 (±42.4)*
CO_2_ use, mean (L)	33 (±30.0)*
Mean intra-abdominal pressure, mmHg (range)	9.9 (8−12)*
Conversion rate, n (%)	1 (1.2 %)
Intra-operative complications, n (%)	2 (2.5 %)
**Post-operative outcomes**	
NRS score, mean (range)	
* At the ward*	1 (0−4)*
* One hour at the ward*	1.8 (0−5)*
* At discharge*	1.4 (0−3)*
Same-day discharge, n (%)	71/80 (88.8 %)*
**Complications 6 weeks post-surgery, n (%)**	4 (4.9 %)
* Clavien-Dindo grade 1*	2
* Clavien-Dindo grade 2*	2
* Clavien-Dindo grade 3a/b*	0
* Clavien-Dindo grade 4*	0
* Clavien-Dindo grade 5*	0
**Re-admission, n (%)**	1 (1.2 %)

NRS = Numeric Rating Scale in pain. SD = standard deviation.

The case in which a conversion occurred was not included in the analyses for the outcomes marked with an *.

In one case, hybrid vNOTES was used in phase 2 due to adhesions of the left adnexa and limited visibility. A hybrid vNOTES procedure combines laparoscopic view through the umbilicus with vNOTES surgery. Adhesiolysis was performed laparoscopically, after which the procedure was completed via vNOTES. This was patient 64, the 24th patient operated on by the surgeon, a 61-year-old with a BMI of 28 kg/m², vaginal parity, and no previous abdominal surgery. The indication was also a risk-reducing bilateral salpingo-oophorectomy due to genetic predisposition.

The mean Numeric Rating Scale (NRS) score upon arrival at the department post-surgery was 1 (range 0–4), after one hour in the department it was 1.8 (range 0–5), and at discharge it was 1.4 (range 0–3). In total, 71 of 80 participants (88.8 %) were discharged the same day. Considering the scheduled SDD, this was applicable for 71 out of 74 participants (95.9 %). Due to the late timing of the surgery (n = 4), one patient undergoing a combined surgery, and one woman without home care, these patients had to stay overnight (Table 2).

#### 3.2.1 Surgical outcomes adjusted for cyst size

In Table 3, surgical outcomes were described based on ovarian cyst size, differentiating between cysts < 5 cm (n = 3) and cysts > 5 cm (n = 12) (Table 3). In the group with cysts < 5 cm, the mean total surgical duration was 39 min (SD 14.5).There were no conversions, and SDD was possible in 66.7 % (n = 2). One patient (33.3 %) had a post-operative complication (Clavien-Dindo grade 1; phlebitis) within 6 weeks, and no re-admissions were observed.

**Table 3:** Surgical outcomes stratified by cyst size.

	**< 5 cm** **(n = 3)**	**> 5 cm** **(n = 12)**	**p-value**
**Intra-operative outcomes**			
Unilateral cyst	3 (100 %)	11 (91.7 %)	-
Bilateral cysts	0 (0 %)	1 (8.3 %)	-
Mean cyst size, cm (SD)	3.9 (±0.2)	9.6 (±4.5)	-
Dermoid cyst, n (%)	0 (0 %)	2 (16.7 %)	-
Mean total duration of surgery, minutes (SD)	39.0 (±14.5)	54.8 (±21.1)	0.250
* Phase 1*	8 (6.0-¥)	7.5 (5.3–10)	0.884
* Phase 2*	23 (14¥)	33.0 (19.8–52.8)	0.083
* Phase 3*	11.7 (±9.3)	8.5 (±2.5)	0.616
Blood loss, mean (mL)	10 (5-¥)	10 (5−10)	0.664
CO_2_ use, mean (L)	35.3 (±35.3)	42.1 (±27.7)	0.722
Mean intra-abdominal pressure, mmHg (range)	10 (-)	9.8 (8−12)	-
Conversion rate, n (%)	0 (0 %)	0 (0 %)	-
Intra-operative complications, n (%)	0 (0 %)	0 (0 %)	-
**Post-operative outcomes**			
Same-day discharge, n (%)	2 (66.7 %)	9 (75 %)*	-
Complications 6 weeks post-surgery, n (%)	1 (33.3 %)^**^	0 (0 %)	-
Re-admission, n (%)	0 (0 %)	0 (0 %)	-

SD = standard deviation. * Three patients requested a day admission.** Clavien-Dindo grade 1.

Normally distributed variables are reported as mean (± SD), with p-values calculated by the Independent Samples t-Test. Non-normally distributed variables are presented as median (IQR), with p-values calculated by the Mann–Whitney *U* test.

¥ Due to the small sample size, the 75th percentile could not be calculated.

In the cysts > 5 cm group, the mean size was 9.6 cm (SD 4.5). The mean total surgical time was 54.8 min (SD 21.1). There were no conversions, with a 75 % SDD rate (n = 9) and no post-operative complications or re-admissions (Table 3). No significant differences were observed between both groups.

#### 3.2.2 Intra-operative and post-operative complications

Among 81 patients, two intra-operative complications (2.5 %) occurred. There was one thermic lesion to the small intestine and one thermic lesion on the rectosigmoid serosa, which were both sutured using vNOTES with a single vicryl 2–0 SH stich. Both patients recovered without complications (Table 4).

**Table 4:** Patient characteristics in the group with intra-operative complications.

**Complication number**	**Age (years)**	**BMI (kg/m**^**2**^**)**	**Indication and procedure**	**Previous abdominal surgery**	**Vaginal delivery**	**Number of surgery for surgeon**	**Complication**	**Recovered by**
1	60	26.8	Gene mutation carrier. Bilateral salpingo-oophorectomy	No	Yes	14th	Thermic lesion to small intestine	Preventively sutured by surgeon
2	44	19.5	Definitive contraception. Bilateral salpingectomy	No	Yes	35th	Thermic lesion to rectosigmoid	Preventively sutured by surgeon

Post-operative complications, as presented in Table 2, within the first six weeks after surgery were observed in 4.9 % (n = 4) of which two Clavien-Dindo grade 1 (phlebitis) and two Clavien-Dindo grade 2 (urinary tract infection). One patient (1.2 %) was readmitted a day post-surgery because of abdominal pain, no cause was found.

## 4 Discussion

Our findings highlight that vNOTES is a safe and feasible technique for a variety of benign adnexal surgeries. In this retrospective cohort study, the mean surgical time was 38.7 min, with one (1.2 %) conversion to laparoscopy, and two (2.5 %) intra-operative complications without re-interventions. In 88.8 % of cases, patients were treated in a day-care setting, with four (4.9 %) post-operative complications within the first 6 weeks. Consistent with our findings, previous research has demonstrated the effectiveness of vNOTES across various indications, including definitive contraception, (prophylactic) bilateral salpingo-oophorectomy, and cystectomy [5], [6], [7], [8]. Compared to conventional laparoscopy, adnexal surgery using vNOTES in both elective and emergency procedures offers advantages, including no abdominal entry-related risks, shorter operative time, reduced hospitalization, decreased post-operative pain, cosmetic benefits for the patient, and ergonomic advantages for the surgeon [5], [13], [14].

The NOTABLE trial highlighted the non-inferiority of vNOTES adnexectomy compared to laparoscopy, with significantly shorter surgery times (24 ± 8 min versus 39 ± 8 min), lower VAS pain scores on day 1 (range 2.0–3.5 versus 4.3–6.0), reduced analgesic use, and SDD in 94 % of cases [4]. There were no conversions in the vNOTES group, one intra-operative complication due to cyst spillage, and four post-operative bleeding complications, one requiring re-intervention [4]. Similar results were found in a paired-sample cross-sectional study by Kaya et al. [13], with vNOTES adnexal surgery being superior compared to conventional laparoscopy, including significantly shorter surgery duration (48.33 ± 33.12 min versus 72.23 ± 43.63 min, p = .04) and reduced postoperative hospital stay (38.4 ± 14.91 h versus 48 ± 17.82 h, p = .03), as well as lower pain scores on VAS at 6 and 24 h postoperatively (respectively 4 (3−7) vs 6 (4−8) p = .003 and 2 (2−3) vs 3 (2−5), p.03) [13].

Surgical time in this cohort study, as in the study of Kaya et al. (38.7 versus 48 min), is longer compared to the NOTABLE trial with an operating time of 24 min. This can be explained by the surgeon’s (J. Baekelandt) experience, having performed over 200 vNOTES procedures before the start of the NOTABLE trial [4]. Rate of SDD in this study was comparable with the NOTABLE trial, respectively 95.9 % eligible and 94 %. vNOTES adnexal surgery shows low rates of conversion and intra-operative complications [5], [7], [8], [13], [14], [15]. In this study, one (1.2 %) conversion to laparoscopy (the 19th patient for the surgeon) and two (2.5 %) intra-operative complications (the 14th and 35th patients) occurred, likely reflecting the early stages of the learning curve. These findings are in line with a recent systematic review by Benton-Bryant (2024), reporting a 1.37 % conversion rate and a 0.22 % intraoperative complication rate in 2261 vNOTES adnexal surgeries [16]. vNOTES adnexal surgery presents technical challenges due to the lack of triangulation, yet evidence suggests that surgeons proficient in both endoscopic and vaginal techniques can achieve competency in cystectomy via vNOTES after approximately 36 cases [15]. To avoid initial technical difficulties, it is recommended to begin with adnexectomy rather than cystectomy [15]. Recent research identifies severe pelvic adhesions, bilateral cysts, and endometriotic cysts as predictors of surgical conversion and longer procedure times [8], [17]. This study showed that in the group with larger cysts, the surgery duration of phase 2 appeared longer. Furthermore, in one case of our study, hybrid vNOTES was utilized due to pelvic adhesions involving the left adnexa, successfully preventing conversion through laparoscopic adhesiolysis in phase 2.

The strength of our study is the relatively large sample size (n = 81) with various benign adnexal indications in both emergency and elective settings. Also, these results reflect the initial performance of gynaecologists which completed the vNOTES hysterectomy learning curve and started with vNOTES adnexal surgery. This is in contrast with most existing literature that emphasize highly experienced surgeons. Therefore, the results of this study can effectively be translated to the general population, particularly for surgeons considering the initiation of vNOTES adnexal surgery.

The limitations of this study include its non-comparative, retrospective design, and single-center setting. Given the retrospective design and the lack of data on patients treated with alternative surgical approaches, the presence of potential selection bias cannot be excluded. Additionally, long-term outcomes remain undetermined. Nonetheless, this study offers valuable insights into the underexplored field of vNOTES adnexal surgery. By expanding the available evidence, we hope to support further investigation and encourage broader adoption of this minimally invasive approach where appropriate.

## Conclusion

This study supports the current findings that vNOTES is a safe and feasible choice for treating benign adnexal pathology. It is a less invasive approach compared to conventional laparoscopic and open surgery with no abdominal scars, a high SDD rate, and low complication rate. Larger prospective and randomized controlled trials will be needed.

## Source of funding

none.

## Clinical trial registration

not applicable.

## IRB Approval

This study was approved by the committee (METCZ20240100, September 3, 2024).

## Prior Presentation

The data of 81 adnexal surgeries have been presented in a poster session at the ESGE congress 2024, Marseille.

## CRediT authorship contribution statement

**Bongers Marlies:** Writing – review & editing. **Wassen Martine:** Writing – review & editing, Visualization, Supervision, Methodology, Investigation, Formal analysis, Data curation, Conceptualization. **Rebecca Henschen:** Writing – original draft, Visualization, Resources, Project administration, Methodology, Investigation, Formal analysis, Data curation, Conceptualization. **Bus Anouk:** Writing – original draft, Visualization, Project administration, Investigation, Formal analysis, Data curation. **Smeets Nicol:** Resources, Investigation, Data curation.

## Declaration of Competing Interest

The authors declare that they have no known competing financial interests or personal relationships that could have appeared to influence the work reported in this article.

## Data Availability

Data is available from the authors upon reasonable request.
